# Modeling Solution Behavior of Poly(*N*-isopropylacrylamide): A Comparison between Water Models

**DOI:** 10.1021/acs.jpcb.2c00637

**Published:** 2022-05-02

**Authors:** Letizia Tavagnacco, Emanuela Zaccarelli, Ester Chiessi

**Affiliations:** † CNR-ISC and Department of Physics, Sapienza University of Rome, Piazzale A, Moro 2, Rome 00185, Italy; ‡ Department of Chemical Sciences and Technologies, University of Rome Tor Vergata, Via della Ricerca Scientifica I, Rome 00133, Italy

## Abstract

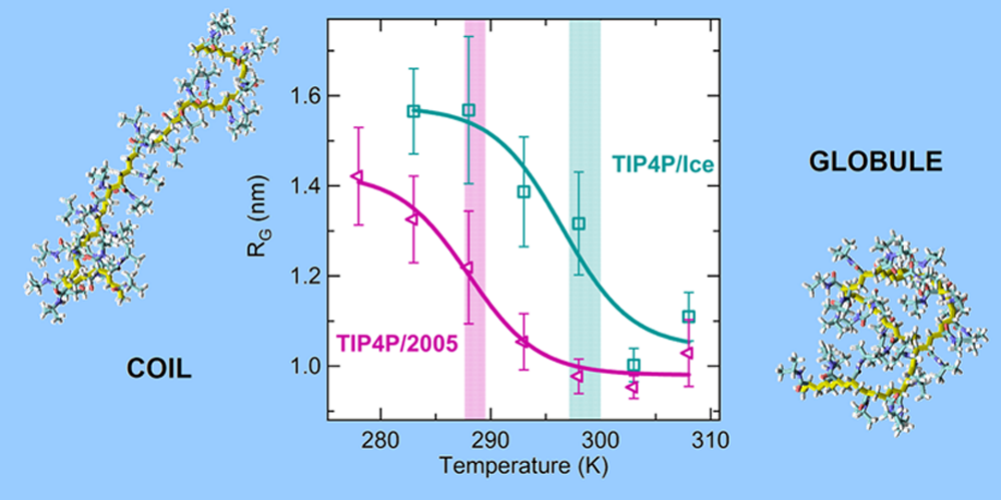

Water is known to play a fundamental role in determining the structure and functionality of macromolecules. The same crucial contribution is also found in the in silico description of polymer aqueous solutions. In this work, we exploit the widely investigated synthetic polymer poly(*N*-isopropylacrylamide) (PNIPAM) to understand the effect of the adopted water model on its solution behavior and to refine the computational setup. By means of atomistic molecular dynamics simulations, we perform a comparative study of PNIPAM aqueous solution using two advanced water models: TIP4P/2005 and TIP4P/Ice. The conformation and hydration features of an atactic 30-mer at infinite dilution are probed at a range of temperature and pressure suitable to detect the coil-to-globule transition and to map the P–T phase diagram. Although both water models can reproduce the temperature-induced coil-to-globule transition at atmospheric pressure and the polymer hydration enhancement that occurs with increasing pressure, the PNIPAM–TIP4P/Ice solution shows better agreement with experimental findings. This result can be attributed to a stronger interaction of TIP4P/Ice water with both hydrophilic and hydrophobic groups of PNIPAM, as well as to a less favorable contribution of the solvent entropy to the coil-to-globule transition.

## Introduction

The temperature- and pressure-induced coil-to-globule transition of poly(*N*-isopropylacrylamide) (PNIPAM) in water has attracted much interest in the scientific community, initially because of its possible analogies with folding processes of natural polypeptides^1,2^ and later for its potential as a mechanism of sophisticated molecular actions, including the mechanical transduction of thermal pulses^3,4^ or the selection of reactants in stimuli-responsive nanodevices.^5−10^ Experimental characterizations focused on mechanistic aspects of the transition, such as the presence of intermediates and hysteresis,^11−14^ highlighting the role of the polymer–water interactions.^15−20^ In particular, the abrupt conformational change from an extended, highly hydrated coil state to a collapsed, partially dehydrated one, triggered by a temperature increase above the coil-to-globule transition temperature (*T*_C_), inspired the idea of a cooperative hydration pattern.^12,16,21−29^ Consequently, access to the molecular details of the PNIPAM chain and of its aqueous surrounding as a function of temperature and pressure, which can be difficult using experimental methods, has gained growing interest. Atomistic molecular dynamics (MD) simulation owes much of its fortune to the successful investigation of biopolymers, as recognized by the Nobel Prize in Chemistry assigned to M. Karplus, M. Levitt, and A. Warshel in 2013.^30^ However, after the start in the biological world, it soon became clear that this computational approach could also help characterize the behavior of synthetic macromolecules,^31,32^ including PNIPAM,^33,34^ in water. In the last decade, several MD simulation studies have been published on PNIPAM with different stereochemistries, degrees of hydration, and slight chemical modifications.^35−62^ These contributions mainly focused on the temperature dependence of the polymer conformation in water and in aqueous mixtures and on its correlation with surrounding solvent molecules.^35−42,44−46,48,49,51,53−58,60^ PNIPAM networks and chain assemblies with a low degree of hydration were also modeled to disclose molecular rearrangements occurring at low temperature^50,52,59^ or in permeation processes.^61^

Some challenges are still open in the atomistic simulation of PNIPAM, concerning model effectiveness and sampling efficiency. In this regard, efforts based on equilibrium MD simulations were coupled with sampling-assisted methods, such as replica exchange molecular dynamics (REMD), metadynamics, and potential of mean force calculations.^48,49,53,55^ The issue of equilibrating PNIPAM conformations with all-atom models has been focused in ref,^54^ where a syndiotactic 30-mer has been simulated at 295 K, by 15 independent 1000 ns trajectory runs, and starting from three different initial configurations. With the used force-field setup, the globule conformation of PNIPAM is preferred at this state point, and the authors show that 1 μs runs, with the equilibration time of the order of 600–700 ns and the remaining time for the production run, are insufficient to reproduce strictly consistent distributions of the radius of gyration. In ref (55), the REMD-enhanced sampling approach is applied to access the *R*_G_ temperature dependence of an atactic PNIPAM 40-mer in water. Moreover, the enthalpy of the coil-to-globule transition is successfully estimated in water and in water/methanol mixtures by means of reproduction and analysis of pools of coiled and globular conformations at 300 K from 200 independent simulations of 20 ns for each solution conditions. This study shows the efficacy of atomistic simulations with assisted sampling approaches, such as REMD, and the need for a wide number of short trajectory replicas to quantitatively determine thermodynamic state functions associated to PNIPAM thermoresponsivity.

Irrespective of the method, the success of the atomistic simulation relies on a suitable choice of the polymer force field and of the water model. As shown in Table 1, a careful literature review detects some heterogeneity in the used PNIPAM force field, especially in older studies,^35,37−40^ and often ad hoc adjustments of partial charges for polymer atoms^42,62^ or of van der Waals polymer–water interactions^56^ are applied to the original force field, in order to reproduce the transition between soluble and insoluble states at the expected temperature.^42,48,49,55,56,62^ Recently, the OPLS-AA force field, with some revisions aimed at increasing PNIPAM–water affinity, has been the one more frequently adopted.^36,40−46,48−52,54,55,57−63^ Interestingly, there is an even greater heterogeneity for the water model selected to simulate this polymer in aqueous environments, including SPC,^35^ SPC/E,^43,45,48,49,53−55,61^ PCFF,^38^ TIP3P,^37,39,56^ TIP4P,^46^ TIP4P/2005,^36,40−42,44,57,58,62^ and TIP4P/Ice^50−52,59,60,63^ water models. With this background and with the aim of extending atomistic simulation methods to other PNIPAM-based systems, it would thus be valuable for the community to identify the best protocol in terms of both PNIPAM and water parametrization. Moreover, as summarized in Table 1, the complex behavior of PNIPAM has been addressed with several different simulation frameworks aiming at understanding distinct physical phenomena; thus, a direct comparison between the available data is not straightforward.

**Table 1:** Overview of Atomistic MD Simulation Studies of PNIPAM since 2011

reference (year)	model system	PNIPAM force field	water force field	thermodynamic region	physical investigation focus
(35)(2011)	oligomer	GROMOS G45A3	SPC	C–G transition	stereoisomerism
(36)(2016)	single chain	OPLS-AA^a^	TIP4P/2005	C–G transition	tacticity
(37)(2012)	oligomers, single chain	OPLS	TIP3P	C–G transition	degree of polymerization
(38)(2012)	oligomers, single chain	PCFF	PCFF	C–G transition	solvation
(39)(2010)	single chain	Amber 94^a^	TIP3P	C–G transition	effect of salt
(40)(2015)	oligomers, single chain	OPLS-AA^a^	TIP4P/2005	C–G transition	PNIPAM hydrophobization
(41)(2017)	chains	OPLS-AA^a^	TIP4P/2005	C–G transition	tacticity
(42)(2016)	oligomer	OPLS-AA^a^	TIP4P/2005	300 K	pressure-induced aggregation
(43)(2016)	single chain, oligomers	OPLS-AA, Amber 94, PCFF	SPCE, TIP3P, PCFF	C–G transition	liquid–liquid phase equilibrium
(44)(2016)	single chain	OPLS-AA	TIP4P/2005	C–G transition	structural changes
(45)(2017)	single chain, membrane	OPLS-AA, AMBER	SPCE	C–G transition	structural changes, effect of salt
(46)(2019)	single chain	OPLS-AA	TIP4P	C–G transition	cononsolvency
(47)(2017)	single chain	OPLS-AA	SPCE	C–G transition	solute adsorption
(48)(2018)	chains	OPLS-AA	SPCE	C–G transition	polymer aggregation
(49)(2018)	single chain	OPLS-AA OPLS-AA^a^	SPCE	C–G transition	thermodynamics, degree of polymerization
(50)(2018)	network	OPLS-AA^a^	TIP4P/ICE	supercooled	dynamical transition
(51)(2018)	single chain	OPLS-AA^a^	TIP4P/ICE	C–G transition	molecular mechanism
(52)(2019)	network	OPLS-AA^a^	TIP4P/ICE	supercooled	dynamical transition
(53)(2019)	single chain	OPLS-AA	SPCE	supercooled, C–G transition	thermodynamics
(54)(2021)	single chain	OPLS-AA	SPCE	295 K	equilibration in solution
(55)(2019)	single chain	OPLS-AA, OPLS-AA^a^	SPCE	300 K	thermodynamics, cononsolvency
(56)(2020)	single chain	CHARMM^a^	TIP3P	C–G transition	*N*-substitution
(57)(2020)	chains, network	OPLS-AA^a^	TIP4P/2005	C–G transition	dynamics
(58)(2020)	single chain	OPLS-AA^a^	TIP4P/2005	C–G transition	cononsolvency
(59)(2021)	chains	OPLS-AA^a^	TIP4P/ICE	supercooled	dynamical transition
(60)(2021)	single chain	OPLS-AA^a^	TIP4P/ICE	C–G transition	cononsolvency
(61)(2020)	single chain, chains	OPLS-AA^a^	SPCE	C–G transition	permeability
(62)(2016)	monomer	OPLS-AA^a^	TIP4P/2005	C–G transition	thermodynamics
(63)(2021)	single chain	OPLS-AA^a^	TIP4P/ICE	C–G transition	effect of pressure

a Parameters or charges modified; C–G transition refers to coil-to-globule transition.

Recent MD simulations provided insights into the behavior of PNIPAM systems in aqueous media,^36,40,41,51,57,58,60^ extending the range of temperatures and pressures to unexplored regions.^50,52,59,63^ These studies have consistently used the same polymer force field, namely, the OPLS-AA^64^ with the modifications of Siu et al.,^65^ but they either employed TIP4P/2005^66^ or TIP4P/Ice^67^ for water, depending on the physical conditions investigated. Both water models, proposed by the Vega’s group in 2005, are very effective in reproducing several water properties, each one with its own peculiarities: on one hand, the TIP4P/2005 model captures very well the dependence of the isothermal compressibility on pressure at 298 K as well as the slope of the P–T coexistence line; on the other hand, the TIP4P/Ice model allows for an excellent prediction of melting temperature. The aim of the present study is to comparatively test the performances of these two water models in conjunction with the modified OPLS-AA force field for PNIPAM^65^ and their ability to reproduce the pressure/temperature behavior of PNIPAM in diluted solution. This comparison, unfeasible from previous studies due to differences in systems and computing protocols, will help define an optimal computing setup for MD simulations of PNIPAM-based systems, as well as of other amphiphilic macromolecules, in aqueous solution. Furthermore, the differences between the conformational behavior of the polymer detected using the two water models in a wide temperature–pressure interval are interpreted on the basis of the different characteristics of these two solvents. This analysis will make it possible to highlight the dominant factors in the hydration pattern, which are needed for a proper molecular description of the coil-to-globule transition of PNIPAM.

## Methods

The pressure–temperature phase diagram of a PNIPAM linear chain in diluted aqueous solution is investigated by performing all-atom MD simulations using two different computational water models, TIP4P/2005^66^ and TIP4P/Ice.^67^ The parameters used to define these models are summarized in Table 2.

**Table 2:** Potential Parameters of the TIP4P/2005 and TIP4P/Ice Water Models^a^

water model	ϵ (K)	σ (Å)	*q*_H_ (e)	*d*_OM_ (Å)	μ (10^–18^ esu cm)	ρ (g cm^–3^)
TIP4P/2005	93.2	3.1589	0.5564	0.1546	2.305	0.9979
TIP4P/Ice	106.1	3.1668	0.5897	0.1577	2.426	0.993

a ϵ and σ are the strength and the size of the Lennard–Jones interaction, respectively; *q*_H_ is the hydrogen site charge; *d*_OM_ is the distance between the oxygen and the *M* site; μ is the dipole moment; and ρ is the density at 298 K and 0.1 MPa from refs (66) and (67).

The polymer chain consists of 30 repeating units, and it is described using atactic stereochemistry, in accordance with the experimental structure.^68,69^ PNIPAM is modeled using a revised version of the OPLS-AA force field,^64^ which incorporates the implementation by Siu et al.^65^ In the simulation protocol, first, an energy-optimized chain^36^ was centered in a cubic box of 8.5 nm side and oriented along the box diagonal to maximize the distance between periodic images. Then, about 22,000 TIP4P/2005 or TIP4P/Ice water molecules were added, and another energy minimization with a tolerance of 1000 kJ mol^–1^ nm^–1^ was carried out. This system was used as the starting configuration of each independent simulation. Trajectories were acquired in the NPT ensemble at six different pressure values, that is, 0.1, 30, 50, 100, 200, and 350 MPa, in a range of temperature between 278 and 308 K. The leapfrog integration algorithm^70^ with a time step of 2 fs was used. Cubic periodic boundary conditions and minimum image convention were applied. The length of bonds involving H atoms was constrained using the LINCS procedure.^71^ The velocity rescaling thermostat coupling algorithm with a time constant of 0.1 ps was used to control temperature.^72^ Pressure was maintained using the Berendsen barostat^73^ using a time constant of 0.5 ps with a standard error on pressure values lower than 10%. The cutoff of nonbonded interactions was set to 1 nm, and electrostatic interactions were calculated using the smooth particle-mesh Ewald method.^74^ Trajectories were acquired for 0.3 μs for each point in the P–T phase diagram using the GROMACS software package (version 2018),^75,76^ and the last 100 ns was considered for analysis, sampling one frame every 5 ps. Independent simulation replica, also using different starting conformations of the polymer chain, were performed at 0.1 MPa and for some critical P–T conditions (Figure S1A–F). Some results of the PNIPAM–TIP4P/Ice simulations are presented in the previous study.^63^

**Figure 1:**
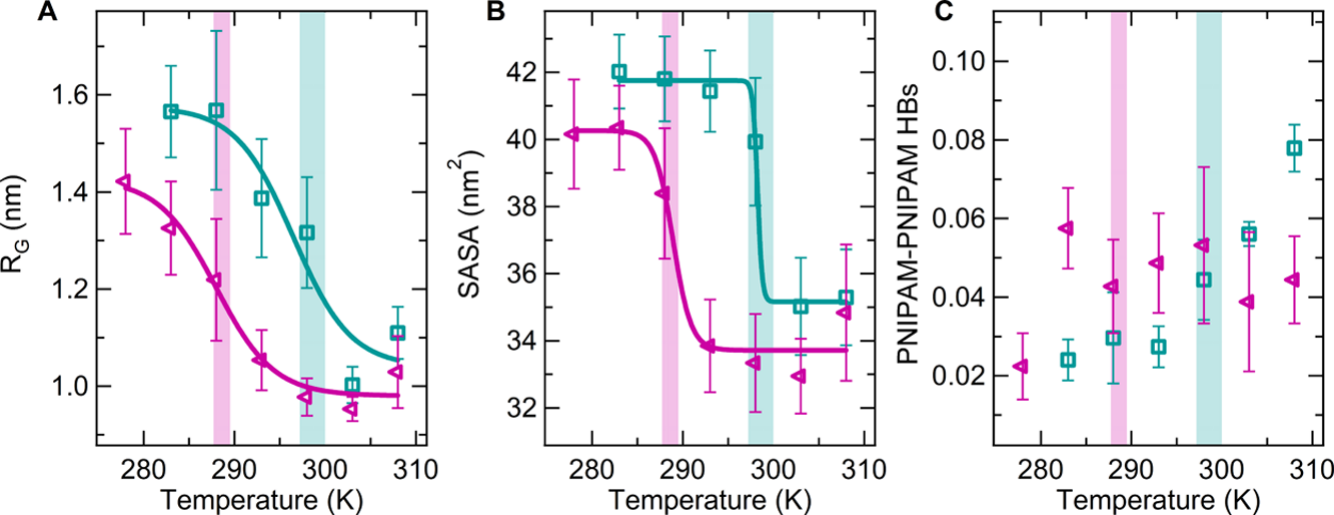
Temperature dependence of (A) PNIPAM radius of gyration *R*_g_; (B) SASA; and (C) total number of PNIPAM–PNIPAM hydrogen bonds, normalized to the number of repeating units, at a pressure of 0.1 MPa for TIP4P/Ice (green squares) and TIP4P/2005 (violet triangles). Data represent time-averaged values over the last 100 ns and their standard deviation. Solid lines are a sigmoidal fit to the data. The transition temperature values, equal to 298.5 ± 1.5 and 288.5 ± 1.0 K for TIP4P/Ice and TIP4P/2005, respectively, are identified by vertical bands including error bars. Data for TIP4P/Ice are reproduced from ref (63).

The polymer chain radius of gyration (*R*_G_) and the solvent accessible surface area (SASA) were used to monitor the occurrence of the coil-to-globule transition. *R*_G_ was calculated through the equation
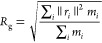
1where *m*_*i*_ is the mass of the *i*th atom and *r*_*i*_ is the position of the *i*th atom with respect to the center of mass of the polymer chain. The SASA was calculated as the van der Waals envelope of the solute molecule extended by the radius of the solvent sphere about each solute atom center.^77^ We used a spherical probe with a radius of 0.14 nm and the values of van der Waals radii of the work of Bondi.^78,79^ Averages were performed over the last 100 ns of trajectory. The transition temperature values at 0.1 MPa were calculated as the average of the *T*_C_ obtained from the sigmoidal fit of the temperature dependence of *R*_G_ and SASA. Globular states were assigned to conformations with an average radius of gyration smaller than 1.2 nm. This cutoff value, already introduced in previous studies,^63^ corresponds to the value of *R*_G_ at the inflection point obtained by fitting using a sigmoidal function the temperature evolution of the average radius of gyration at atmospheric pressure (Figure 1A).

To analyze the interactions occurring between the polymer and water, hydrogen bonds were evaluated by adopting the geometric criteria of an acceptor–donor distance lower than 0.35 nm and a hydrogen-donor–acceptor angle lower than 30°. Polymer hydration was also characterized by defining water molecules in the first solvation shell as the molecules having the water oxygen atom at a distance from PNIPAM nitrogen/oxygen atoms lower than 0.35 nm (hydrophilic water molecules) or a distance from methyl carbon atoms of PNIPAM lower than 0.55 nm (hydrophobic water molecules). The fraction of hydration water molecules at the interface with hydrophobic groups (F) was defined as the number of water molecules in the first hydration shell of methyl groups divided by the total number of water molecules in the PNIPAM first hydration shell. Moreover, hydrogen bonds between water molecules of the first solvation shell and in the bulk were also analyzed with the same geometric criteria. Hydrated globular states were defined on the basis of a cutoff value of 12.5 hydration water molecules per PNIPAM repeating unit. In analogy with the definition of globular conformations, this cutoff value corresponds to the number of hydration of water molecules per PNIPAM residue at the inflection point obtained by fitting using a sigmoidal function the temperature evolution of the average number of hydration water molecules at 0.1 MPa.

As far as the issue of equilibrating PNIPAM conformations with all-atom models is concerned, using the simulation protocol of the present work, we adopt a compromise between a sustainable computing cost and a full sampling of conformations. The main motivation of this study is the comparison between two advanced water models, one of them being largely used in simulations of polymers and biomacromolecules, in terms of their ability to reproduce the temperature and pressure dependence of PNIPAM conformations in aqueous solutions. In this respect, we assume that a possible systematic error for sampling deficiency would similarly affect the simulations in TIP4P/ICE and TIP4P/2005, for the similarity of these models in water molecule representation, thus preserving the comparative character of this contribution.

## Results and Discussion

To illustrate the comparison between the results obtained with the TIP4P/2005 and TIP4P/Ice models, we first analyze the conformational behavior and solvation of PNIPAM as a function of temperature at atmospheric pressure. Then, we discuss the temperature-dependent evolution of the system at higher pressures up to 350 MPa, and finally, we summarize the temperature–pressure influence on the conformation and hydration of PNIPAM to draw an approximate phase diagram of the solution.

### Coil-to-Globule Transition at 0.1 MPa

Here, we focus on the ability of the simulation setup to properly detect, at atmospheric pressure, the temperature-induced coil-to-globule transition, that is, the single chain process associated to the phase separation of PNIPAM aqueous solution occurring at the lower critical solution temperature (LCST).^11,12^ As diagnostic observables, we monitor the radius of gyration (*R*_G_) and the SASA of the polymer chain, whose behavior in the temperature interval 278–308 K is displayed in Figure 1A,1B.

By increasing temperature, in both TIP4P/2005 and TIP4P/Ice solutions, we observe a regular evolution toward conformational states with smaller size and water interface, which is expected according to the experimental chain behavior.^11,12^ This allows us to estimate using a sigmoidal fit the value of the coil-to-globule transition temperature, equal to 288.5 ± 1.5 and 298.5 ± 1.5 K for the PNIPAM solutions with TIP4P/2005 and TIP4P/Ice water models, respectively, as confirmed in an independent simulation replica (see Figure S1A,B). Considering the experimental *T*_C_ value, of about 305 K,^12^ the TIP4P/Ice water model provides a better description. Moreover, a clear difference between the two models emerges from Figure 1A,B, which shows the inability of TIP4P/2005 water simulations to sample very extended conformations of the PNIPAM chain, even in the temperature range where coiled conformations should be favored. This finding is further illustrated in Figures S2 and S3, where the time evolution and the distribution of values of the polymer radius of gyration are reported. The underestimations of both the *T*_C_ value and of the chain size are consistent with each other, demonstrating a lower water affinity of PNIPAM in TIP4P/2005 solution than in the TIP4P/Ice one. Therefore, the TIP4P/2005 water appears to be a less “good solvent” for PNIPAM, with the consequence of a partial reduction of the structural gap between coil and globule states. Accordingly, the simulations using this water model cannot detect the variation of the intrachain hydrogen bonding as a function of temperature, whereas this is observed in simulations using the TIP4P/Ice water model (Figure 1C).

**Figure 2:**
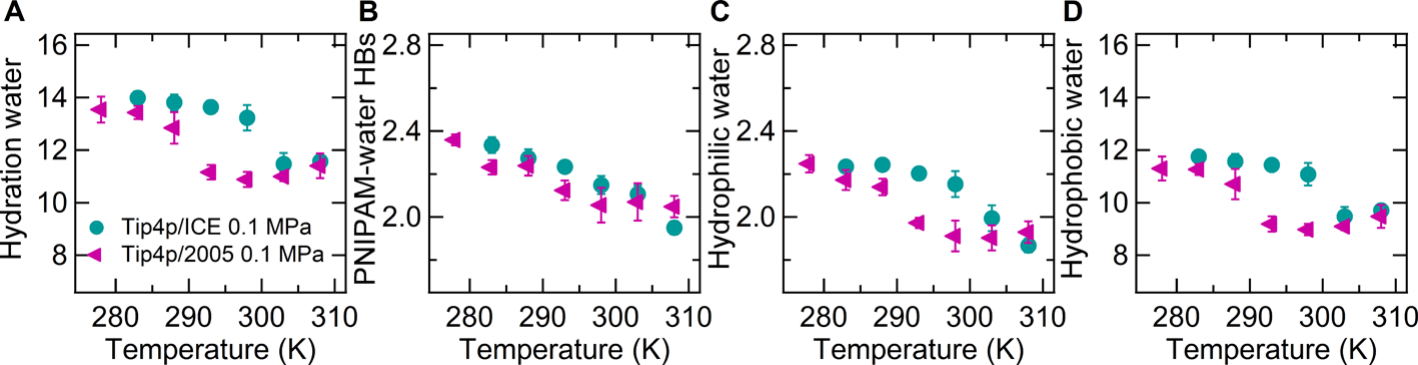
Temperature dependence of (A) number of hydration water molecules; (B) number of PNIPAM–water hydrogen bonds; (C) number of hydrophilic water molecules; and (D) number of hydrophobic water molecules normalized to the number of repeating units and averaged over the last 100 ns of simulation. Data are calculated at 0.1 MPa for TIP4P/Ice (green circles) and TIP4P/2005 (violet triangles). Data for TIP4P/Ice are reproduced from ref (63).

We now turn our attention to the properties of hydration water molecules by analyzing those close to hydrophilic or hydrophobic PNIPAM groups and by monitoring the formation of hydrogen bonding interactions, as reported in Figure 2A–D. The temperature dependence of the number of hydration water molecules is directly correlated to the conformational transition of the polymer and follows the same trend as the radius of gyration. The analysis of the polymer solvation confirms the previous conclusions on the quality of the solvent. The number of hydration water molecules found for the TIP4P/2005 model is lower than the one occurring in the TIP4P/Ice model at all temperatures (Figure 2A), including the state point at 283 K, which for both models corresponds to a stable coil state. In addition, the correlation between *R*_G_ and the number of hydration water molecules at 283 K is displayed in Figure S4A, showing that the lower *R*_G_ values sampled in the TIP4P/2005 water solution correspond to a lower hydration degree of the polymer.

Considering the parametrization of the two water models and the more polar character of the TIP4P/Ice molecule (Table 2), the higher solubility of the polymer, detected using this model, could be attributed to a stronger interaction with polar groups of PNIPAM. Indeed, the water–polymer hydrogen bonding is generally higher for TIP4P/Ice than for the TIP4P/2005 model (Figure 2B), as well as the number of water molecules in the vicinity of amide groups (Figure 2C). However, the hydration of hydrophobic regions of PNIPAM is also larger in the TIP4P/Ice solution, especially at the intermediate temperatures (Figure 2D). The higher affinity of this water model toward apolar groups is demonstrated by the hydration free energy of methane calculated in TIP4P/Ice, which is in excellent agreement with the experimental value and found to be lower than the corresponding hydration free energy obtained in TIP4P/2005 water.^80^ Therefore, TIP4P/Ice water is found to be a better solvent for both polar and apolar PNIPAM components. Hydrophobic hydration has been proposed as having a direct^29^ role and an indirect^81^ role in the solution behavior of PNIPAM. The study of Ono and Shikata hypothesized the formation of water-water hydrogen bond bridges between adjacent isopropylamide groups, directly sustaining extended polymer conformations below the LCST.^29^ According to Bischofberger et al.,^81^ the solubility of PNIPAM in an aqueous environment is influenced by the hydration/dehydration of hydrophobic moieties because of the different chemical potential of water in the polymer hydration shell and in the bulk of the solution. In light of these experimental suggestions, we analyzed the weight of the hydrophobic component in the polymer hydration, looking for differences between TIP4P/Ice and TIP4P/2005 simulations.

**Figure 3:**
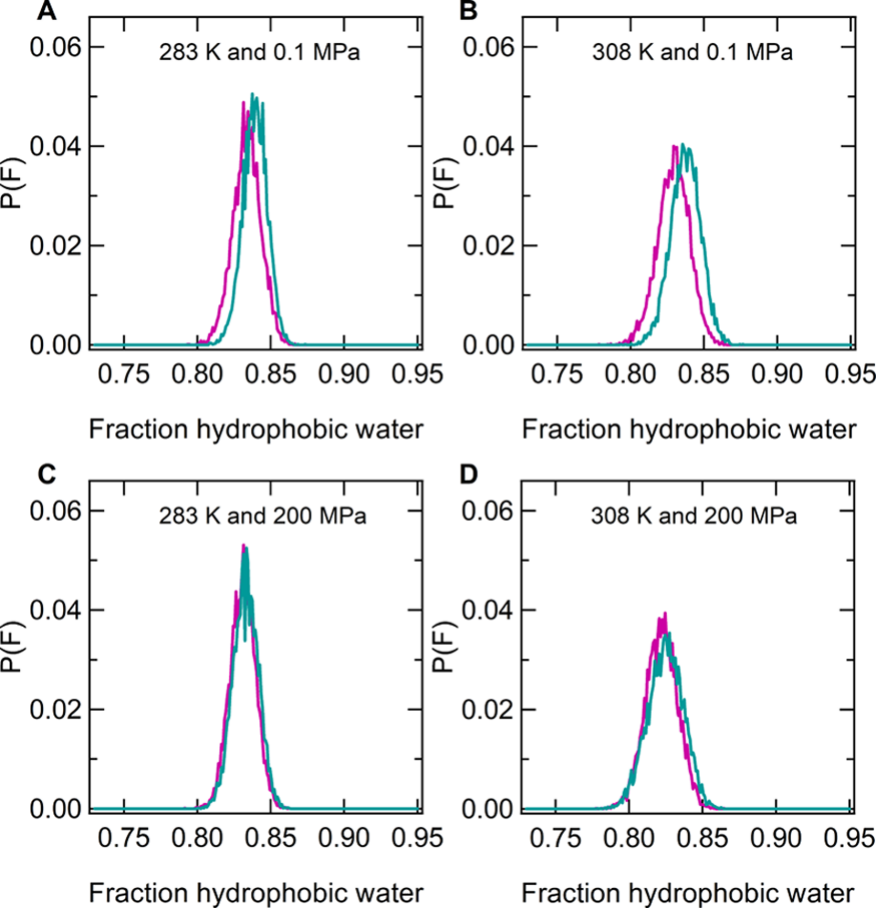
Distribution of values of the fraction of hydrophobic water molecules (F) calculated over the last 100 ns of the simulation data at a pressure of 0.1 MPa and temperature values of (A) 283 and (B) 308 K and at a pressure of 200 MPa and temperature values of (C) 283 and (D) 308 K for TIP4P/Ice (green) and TIP4P/2005 (violet), respectively.

Figure 3A,B shows the distribution of the values of the fraction of hydration water molecules at the interface with hydrophobic groups (F) at atmospheric pressure and compares them to those at 200 MPa (Figure 3C,D), at both 283 and 308 K, where coil and globular states are stable, respectively. F values are greater than 0.8, indicating that hydration of hydrophobic groups is the major component for PNIPAM. As a common feature of the two water models, the distributions are larger for the globular states than for the coil states, which is attributable to the higher thermal agitation. However, the distribution maxima do not depend on temperature and thus on chain conformation. This finding indicates that although in the globular state, the aggregation of hydrophobic moieties occurs with a consequent decrease of the total hydrophobic interface area with respect to the coil state, a similar relative decrease involves the hydrophilic interface area. The distributions obtained at 0.1 MPa in the TIP4P/Ice solution (Figure 3A,B) are slightly shifted to larger abscissa values, that is, the weight of the hydrophobic hydration is higher for this model than for TIP4P/2005. Significantly, this hydration mode characterizes the water model with the better performance in reproducing PNIPAM coil-to-globule transition at atmospheric pressure. Furthermore, we comparatively analyzed the connectivity between water molecules composing the first hydration shell of PNIPAM and in the bulk of the solution at 283 and 308 K. The number of hydrogen bonds per water molecule in these two domains is reported in Table 3, for both TIP4P/Ice and TIP4P/2005 simulations. Irrespective of the domain and at the same conditions, the TIP4P/Ice water molecules form more hydrogen bonds than TIP4P/2005 water molecules. Considering the state at 283 K, the higher hydrogen bonding connectivity of TIP4P/Ice water molecules in the PNIPAM hydration shell contributes to the stabilization of extended conformations, as proposed in ref (29). Therefore, the increased solubility of PNIPAM in TIP4P/Ice water is supported by both increased dipolar and hydrogen bonding interactions between the polymer and water and by a more connected and hydrophobic interfaced water shell.

**Table 3:** Hydrogen Bonding of PNIPAM Hydration and Bulk Water at 0.1 MPa^a^

	bulk water		hydration water	
*T* (K)	TIP4P/Ice	TIP4P/2005	TIP4P/Ice	TIP4P/2005
283	1.813 (±0.003)	1.773 (±0.002)	1.322 (±0.005)	1.279 (±0.009)
308	1.777 (±0.001)	1.726 (±0.002)	1.275 (±0.003)	1.221 (±0.010)

a Average number of water–water hydrogen bonds per water molecule, with standard deviation.

On the basis of the features of PNIPAM solvation in the two water models, we now hypothesize an explanation for the large difference of the *T*_C_ value detected in TIP4P/Ice and TIP4P/2005 solutions. Figure 2A clearly shows the occurrence of an abrupt dehydration of PNIPAM in both solutions when temperature is increased, concerted to the coil-to-globule transition (Figure 1). However, in TIP4P/2005 water, this process is anticipated by about 10 K. Although in absolute values, the hydration degree of PNIPAM is greater in TIP4P/Ice than in TIP4P/2005 solution, the variations of the hydration shell and of the PNIPAM–water hydrogen bonding at the transition are similar in the two systems (Figure 2). In particular, at *T*_C_, the same number of water molecules is released from the hydration shell to the bulk, experiencing a change in hydrogen bonding. In addition, according to the results reported in Table 3, TIP4P/Ice and TIP4P/2005 water molecules undergo the same increase of hydrogen bonding, equal to 0.5 hydrogen bonds per water molecule, moving from the polymer shell to the bulk region. Overall, these characteristics suggest that the coil-to-globule transition enthalpy (Δ*H*_c–g_), which is positive and mainly determined by changes in polymer–water and water–water interactions, has a similar value in the two solutions. Therefore, the lower *T*_C_ value for PNIPAM in TIP4P/2005 water should be ascribed to a higher transition entropy (Δ*S*_c–g_), being *T*_C_ = Δ*H*_c–g_/Δ*S*_c–g_. The main physical factor determining a positive Δ*S*_c–g_ value in this kind of conformational transition of amphiphilic macromolecules is the decrease of the excluded volume to water, which leads to a concomitant increase of the translational entropy of solvent molecules.^82^ However, this gain of translational entropy is related to the solvent density and increases at higher density since a larger number of water molecules acquires translational degrees of freedom.^83^ By comparing the water density values of TIP4P/Ice and TIP4P/2005 at room temperature (Table 2), we note that the latter model has a higher density, leading to the conclusion that a higher coil-to-globule transition entropy results in the lower value of *T*_C_ for PNIPAM in the TIP4P/2005 solution.

### Solution Behavior above Atmospheric Pressure

In our simulations, we scanned the phase diagram of PNIPAM aqueous solution by changing the temperature under different isobaric conditions from 0.1 to 350 MPa, within a temperature interval suitable to cross the transition temperature. In this pressure range, the experimental P–T phase diagram of PNIPAM aqueous solution is characterized by a nonmonotonic phase separation curve, where the transition temperature increases for pressures from 0.1 to about 50–100 MPa and then decreases at higher pressure.^84−87^ Above about 200 MPa, phase separation takes place at lower temperatures as compared to atmospheric pressure. However, a Fourier transform infrared (FTIR) spectroscopy investigation^88^ detected a general enhancement of amide group hydration in the high-pressure regime. Moreover, a peculiar rehydration of PNIPAM microgels in the collapsed state has been observed at pressures of 100 MPa and over, using small-angle X-ray scattering and FTIR measurements.^89,90^ Therefore, although both temperature and pressure can induce the PNIPAM transition to a collapsed state in linear and crosslinked systems,^91,92^ these variables act antagonistically, and pressure can only partially compensate for the thermally induced transition.^89^

**Figure 4:**
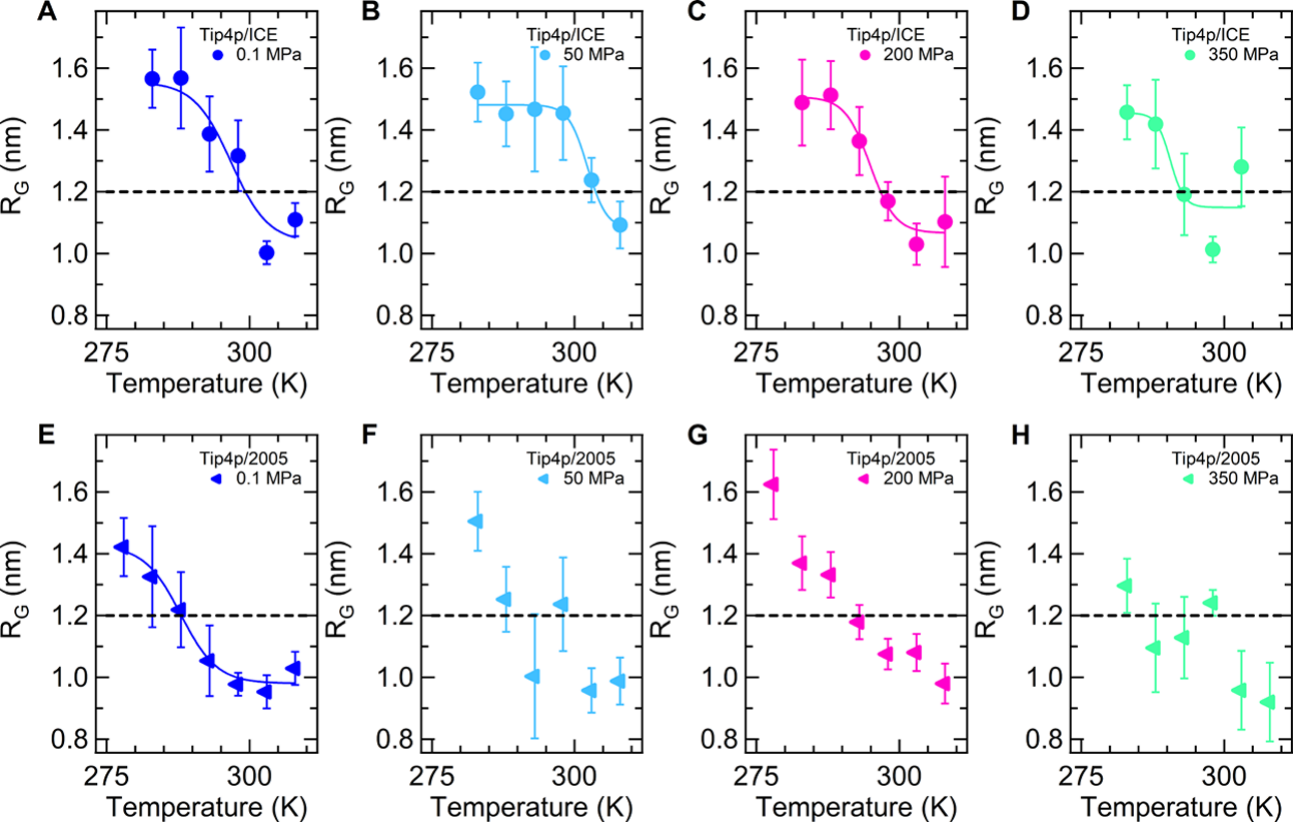
Temperature dependence of PNIPAM radius of gyration at pressure values of (A,E) 0.1, (B,F) 50, (C,G) 200, and (D,H) 350 MPa for TIP4P/Ice (circles) and TIP4P/2005 (triangles). Data represent time-averaged values over the last 100 ns and standard deviation. Solid lines are the sigmoidal fit to the data. The dashed horizontal lines mark the critical value used to define collapsed conformations. Some data for TIP4P/Ice are reproduced from ref (63).

The temperature behavior of *R*_G_ at different pressures is shown in Figure 4A–H, comparing the results of TIP4P/Ice and TIP4P/2005 solutions. For the discussion of these findings, we apply the operative criterion of considering *R*_G_ = 1.2 nm as the threshold to discriminate between coil and globule states. In the PNIPAM–TIP4P/Ice system, *R*_G_ shows a uniform decreasing trend with temperature at all pressures, allowing us to distinguish the temperature stability ranges of coil and globular states. Figure 4A–D highlights the initial increase and subsequent decrease of the temperature where the threshold *R*_G_ value is crossed, changing the pressure from 0.1 to 350 MPa, similarly to the experimental phase behavior. Differently, the isobaric behavior of *R*_G_ at 50, 200, and 350 MPa as a function of temperature for the PNIPAM–TIP4P/2005 system (Figure 4F–H) is more irregular, with oscillations between coil and globule states with increasing temperature. In general, globular conformations are sampled at lower temperatures, as compared to the corresponding isobaric temperature scans in TIP4P/Ice water. At 350 MPa, the chain mainly populates globular states except at 283 K, and extremely small *R*_G_ values are observed at higher temperatures (Figure 4H).

We next consider the hydration features of coil and globule states in the high-pressure regime that can be used to monitor the conformational changes of the polymer system. The correlation between *R*_G_ and the number of hydration water molecules for PNIPAM in TIP4P/Ice and TIP4P/2005 solutions at 200 MPa and 283 K, a condition for which coiled states are sampled using both water models, is illustrated in Figure S4B. A higher hydration degree of the polymer chain, as compared to that at 0.1 MPa, can be observed at 200 MPa at this temperature in the two systems, in agreement with experimental findings.^89^ As a further characteristic of the solvation, we analyzed the fraction of hydrophobic hydration water, whose distribution of values at 200 MPa is shown in Figure 3C,D at 283 and 308 K, respectively. Unlike what detected at 0.1 MPa (Figure 3A,B), no difference between the two water models emerges for this property. However, the comparison with the findings of Figure 3A,B highlights a change of the PNIPAM hydration pattern in TIP4P/Ice water moving from 0.1 to 200 MPa, with a higher contribution of hydration of hydrophilic groups at the higher pressure, as proposed in ref (88). A similar change is not observed for the TIP4P/2005 solution.

The temperature behavior of the number of hydration water molecules at 50, 200, and 350 MPa is reported in Figure S5A–D of the Supporting Information. At 50 MPa, the hydration degree of the polymer chain is quite similar to that observed at atmospheric pressure and higher in TIP4P/Ice water. At higher pressures, PNIPAM is surrounded by a larger number of hydration water molecules, as compared to that at atmospheric pressure, in both polymer solutions at all temperatures, as already pointed out for the condition at 200 MPa and 283 K. However, the water affinity of PNIPAM remains higher in the TIP4P/Ice solution also at high pressures, with the number of solvent molecules of the polymer hydration shell being in general greater in TIP4P/Ice than in TIP4P/2005 (Figure S5C,D). We now focus on the polymer hydration at 350 MPa and 303 K: in the P–T phase diagram, this condition identifies a phase-separated state,^86^ corresponding for the single chain to a globule state. Accordingly, globular conformations are sampled by the simulation using both water models (Figure 4D–H). However, experimental studies document an enhancement of polymer hydration at high pressure above the transition temperature,^89^ also detected using the TIP4P/Ice water model.^63^ By considering the results of Figure S5D, the TIP4P/2005 water model seems to be unable to fully reproduce this feature since the number of hydration water molecules has a very low value at 350 MPa and 303 K, and it further decreases at 308 K.

**Figure 5:**
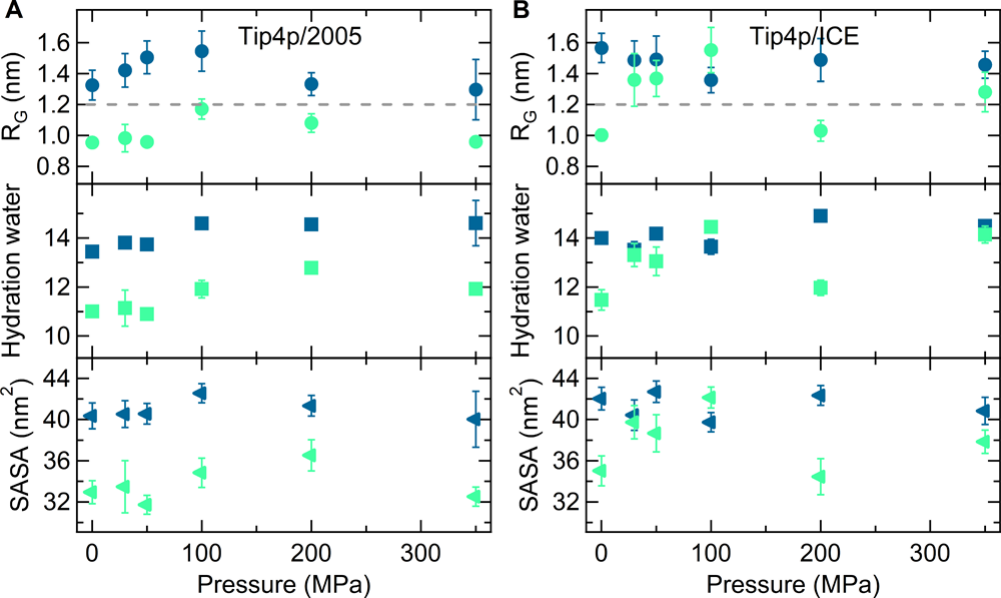
Pressure dependence of the average radius of gyration (circles); the total number of hydration water molecules normalized to the number of repeating units (squares); and the SASA (triangles) calculated for (A) TIP4P/2005 and (B) TIP4P/Ice at *T* = 283 (blue) and 303 (green) K. The dashed horizontal lines mark the critical value used to define collapsed conformations.

**Figure 6:**
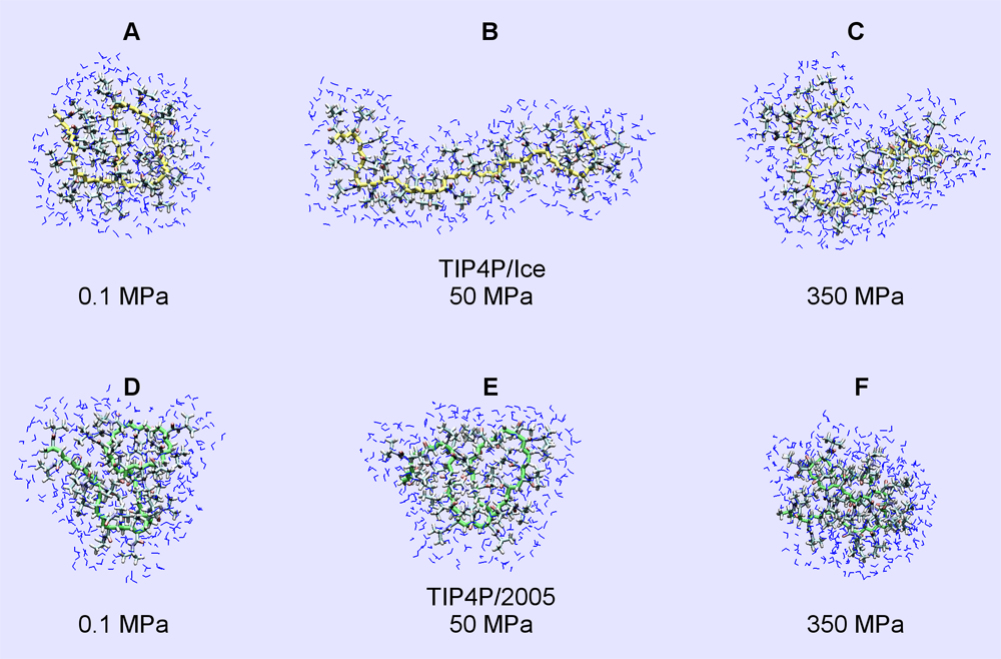
Comparative snapshots of a PNIPAM chain simulated at 303 K in TIP4P/Ice at pressure values of (A) 0.1, (B) 50, and (C) 350 MPa and in TIP4P/2005 at pressure values of (D) 0.1, (E) 50, and (F) 350 MPa. PNIPAM backbone carbon atoms are shown in yellow/green for the TIP4PICE/TIP4P2005 water models, respectively. Hydrogen, carbon, oxygen, and nitrogen atoms are shown in gray, light blue, red, and blue, respectively. Hydration water molecules are displayed in blue.

To highlight the influence of pressure on the conformation and hydration of PNIPAM, we show in Figure 5A,B the behavior of *R*_G_, SASA, and the number of hydration water molecules as a function of pressure for two isotherms: the first one is chosen at 283 K, which in the experimental phase diagram corresponds to a region of stability of the one-phase solution up to extremely high pressures, while the second temperature is 303 K, close to the LCST and to the coil-to-globule transition temperature.^11,12^ In our simulations, the *T*_C_ value at 0.1 MPa is higher than 283 K for both solution models (Figure 1A), and at this temperature, coil states are preferred at all investigated pressures, consistent with the experimental behavior. At 303 K and 0.1 MPa, PNIPAM is in the globule state, again both in TIP4P/Ice and in TIP4P/2005 water. However, at 303 K, the evolution of the system with increasing pressure is different for the two solution models. In TIP4P/Ice water, we detect a significant increase of chain size and hydration up to 100 MPa with a subsequent decrease at higher pressures, which qualitatively agrees with the re-entrant behavior occurring for an isothermal increase of pressure at a temperature slightly above the transition temperature at 0.1 MPa.^93^ On the other hand, in the TIP4P/2005 solution at 303 K, the observables monitored in Figure 5B display a more attenuated dependence on pressure, with the globule state always remaining the preferred conformation. The discrepancies between the conformational features and hydration of PNIPAM in TIP4P/Ice and TIP4P/2005 water as a function of pressure at 303 K are illustrated in Figure 6A–F. In the TIP4P/2005 solution, the redissolution of the polymer chain at intermediate pressure and the formation of a largely hydrated collapsed state at high pressure are not detected within our simulations. In general, it appears that in TIP4P/2005 water, the pressure increase has a more limited effect on the conformation and hydration of PNIPAM globular states, as compared to what is observed in TIP4P/Ice water (Figure 5A,B).

To explain this difference, we examined the pressure dependence of the solvent density, which is found to be similar for both water models in the investigated temperature interval.^94,95^ Hence, the reason for the discrepancy should be ascribed to a different interaction with the polymer. A simulation study of the melting of methane hydrate has highlighted a lower stability of the TIP4P/2005 water interaction with methane, as compared to that of TIP4P/Ice, even above atmospheric pressure.^96^ Therefore, a weaker interaction of water with the apolar groups of PNIPAM, which consequently results in the stabilization of intrachain hydrophobic contacts, could be responsible for the attenuated hydration and conformation response to pressure occurring in the TIP4P/2005 solution. It is noteworthy that the destabilization of the interactions between hydrophobic groups and their rehydration is considered to be a factor in the pressure-induced denaturation of proteins,^97,98^ a process analogous to the pressure-induced globule-to-coil transition of PNIPAM.

**Figure 7:**
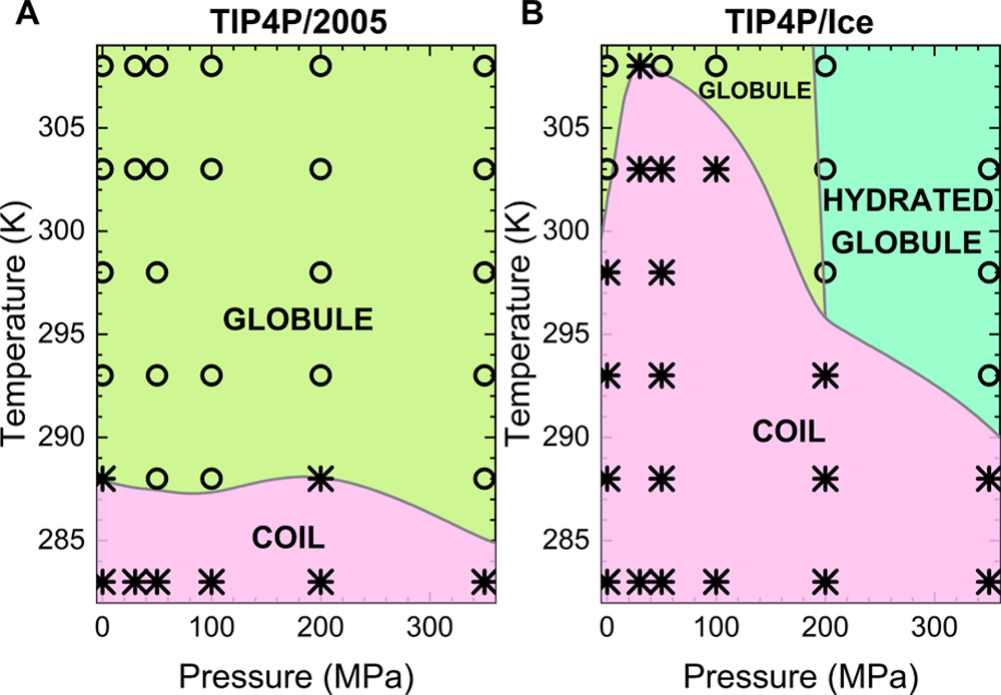
PNIPAM phase diagram in (A) TIP4P/2005 and (B) TIP4P/Ice water. State points where coil and globule conformations are populated are shown with asterisks and circles, respectively. The threshold of *R*_G_ = 1.2 nm was used to distinguish coil and globule states. Hydrated globules are characterized by a number of hydration water molecules/residues equal to or greater than 12.5. Colored areas and phase boundaries are guides to the eye. Some data for TIP4P/Ice are reproduced from ref (63).

Overall, these findings suggest that using the TIP4P/2005 water model, some features of the influence of temperature and pressure on PNIPAM aqueous solution can be qualitatively reproduced: (i) the thermally induced coil-to-globule transition at constant pressure and (ii) the effect of pressure on the polymer hydration, especially at low temperatures. However, the balance between temperature and pressure effects is not adequately captured, leading to discrepancies of the phase boundaries as compared to what is obtained using the TIP4P/Ice model.^63^ An approximate P–T phase diagram for the PNIPAM–TIP4P/2005 solution is drawn in Figure 7A, including the results of additional simulations at 30 and 100 MPa (discussed in Table S1). State points are associated to coil and globule conformations on the basis of the average radius of gyration of the polymer chain, using a cutoff value of 1.2 nm, as defined in the Methods section. To directly compare the simulations results with the experimental observation of rehydration of PNIPAM microgels in the collapsed state at high pressure,^89,90^ a further distinction between globular and hydrated globular states is obtained using the criterion based on the number of hydration water molecules, being higher than 12.5 for the latter (see the Methods section). Phase boundaries are estimated on the overall trend of the defined state points and represent a guide to the eye due to the finite number of examined conditions. In the resulting phase diagrams reported in Figure 7A,B, we note that using the TIP4P/2005 water model, the hydrated globule state is not detected for any of the explored pressure/temperature parameters, in qualitative disagreement with experiments. To test the arbitrariness of the chosen cutoff values, we have additionally explored the changes observed in the phase diagram when using a different cutoff value of *R*_G_ = 1.1 nm to define globular states. Notwithstanding the use of a different cutoff value, the overall position and shape of the phase separation curve between coil and globule states in Figure 7A do not appreciably change, supporting the validity of the adopted criteria.

## Conclusions

The single-chain behavior of PNIPAM in water is dictated by a delicate balance between conformational constraints and polymer–water and water–water interactions. To these factors, the topology of the PNIPAM–solvent interface and its variations with the polymer conformation add an entropic contribution related to the translational motion of water molecules. In this work, we focused on the comparison between two advanced water models, TIP4P/2005 and TIP4P/Ice, in reproducing the main features of the PNIPAM coil-to-globule transition within the same computational setup conditions and the same force field for the polymer. Our results altogether show that the characteristics of the TIP4P/2005 water model lead to an underestimation of the PNIPAM water affinity, as compared to the TIP4P/Ice water model, thereby reducing the thermodynamic stability domain of the coil state. In particular, at atmospheric pressure, the transition to a globular state is anticipated at a lower temperature, by roughly 10 K, and the phase boundary in the P–T diagram has an approximately flat behavior, lacking the details of the *T*_C_ dependence on pressure. We thus postulate that the weaker interaction of TIP4P/2005 water with polymer nonpolar groups, as compared to the TIP4P/Ice model, contributes to the lower sensitivity to pressure of PNIPAM globular conformations. The better agreement with experimental results obtained for TIP4P/Ice water at 0.1 MPa can be explained with a lower coil-to-globule transition entropy due to the lower density of this water model. Based on this hypothesis, it is possible that the efficiency of the TIP4P/2005 water model in describing the PNIPAM solution behavior could improve in simulations using larger polymer models and/or more concentrated solutions. In these systems, the variation of the solvent-excluded volume per repeating unit at the transition is smaller, as compared to a 30-mer at infinite dilution, and the distinction between the results in TIP4P/2005 and TIP4P/Ice water should then decrease. This aspect should thus be explored in future studies. In summary, although the TIP4P/2005 model is often regarded as the best available option close to room temperature, our work provides evidence that in order to provide an adequate description of PNIPAM affinity to water, it is preferable to work using the TIP4P/Ice model across the whole investigated temperature range. This choice appears to be even more crucial to appropriately model the pressure response of the solution, particularly at high pressures, where the TIP4P/2005 model fails to reproduce the more hydrated globular state observed for the TIP4P/Ice model and in experiments.

## Supporting Information Available

Reproducibility of single trajectory data, hydration of PNIPAM in TIP4P/2005 and TIP4P/Ice, and PNIPAM–TIP4P/2005 phase diagram data (PDF)
