# Supplementary material for: Modeling Solution Behavior of Poly(N-isopropylacrylamide): A Comparison between Water Models
Source: J Phys Chem B. 2022 May 2;126(20):3778–88. doi: 10.1021/acs.jpcb.2c00637 (PMC9150113; doi:10.1021/acs.jpcb.2c00637)
Supplement: Supplementary file 1 — jp2c00637_si_001.pdf [file jp2c00637_si_001.pdf]

Supporting Information for 'Modeling Solution Behavior of Poly(N-isopropylacrylamide): a Comparison Between Water Models'

Letizia Tavagnacco,[1,*] Emanuela Zaccarelli,[1,†] and Ester Chiessi[2,‡]

[1] *CNR-ISC and Department of Physics, Sapienza University of Rome, Piazzale A. Moro 2, 00185, Rome, Italy.*

[2] *Department of Chemical Sciences and Technologies, University of Rome Tor Vergata, Via della Ricerca Scientifica I, 00133 Rome, Italy.*

I. REPRODUCIBILITY OF SINGLE TRAJECTORY DATA

Independent simulation replica were performed at 0.1 MPa and for some critical P/T conditions also with different starting chain conformations to test the reproducibility of simulation data. The results are summarized in Figure S1. In addition, the time evolution of PNIPAM radius of gyration in the whole trajectories collected in TIP4P/2005 is reported in Figure S2. To verify that by using a criterion based on the average radius of gyration a correct distinction between coil and globule states can be obtained, Figure S3 summarizes the distribution of radius of gyration as a function of temperature for some representative state points.

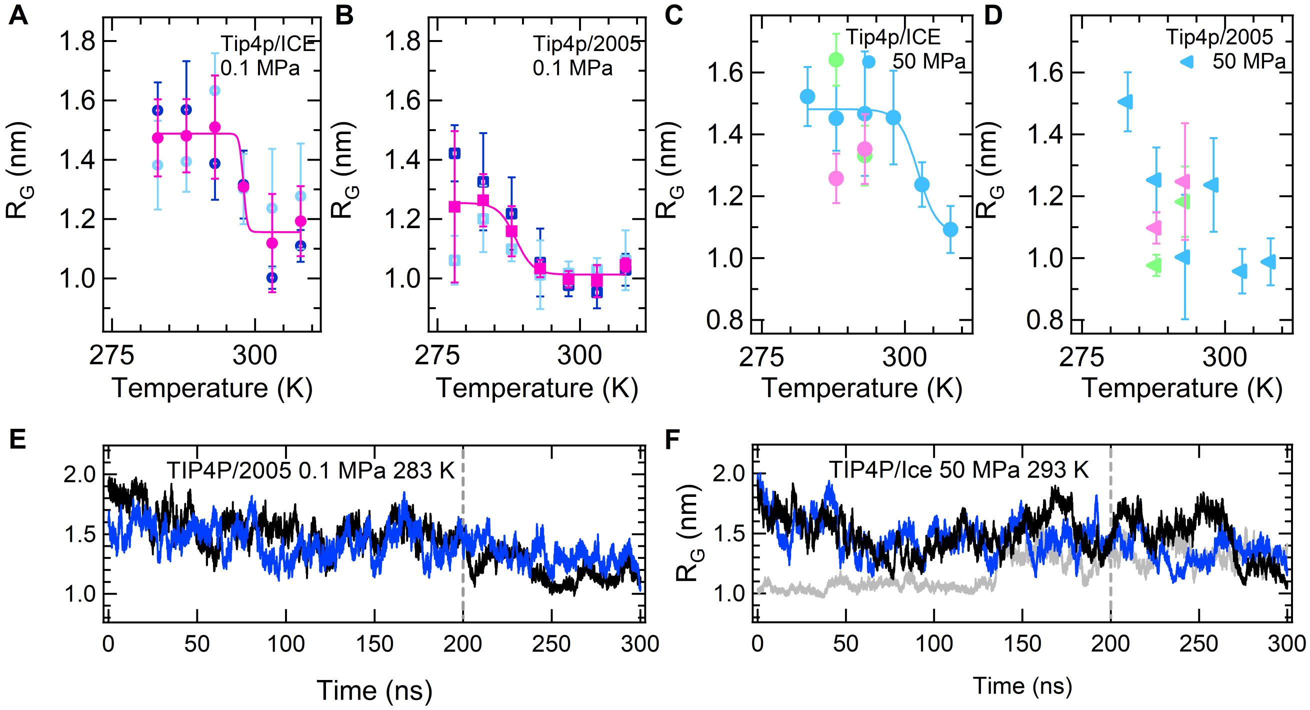

FIG. S1. Temperature dependence of PNIPAM radius of gyration at pressure values of 0.1 MPa for (A) TIP4P/Ice and (B) TIP4P/2005 and at pressure values of 50 MPa for (C) TIP4P/Ice and (D) TIP4P/2005. Data represent time averaged values over the last 100 ns and standard deviation. At 0.1 MPa simulations collected in the Replica 1, 2 and the average of the two replica are shown in blue, light blue, and pink, respectively. At 50 MPa, two independent replica are reported at 288 and 293 K in pink and green. Time evolution of PNIPAM radius of gyration with different starting chain conformations for TIP4P/2005 at 0.1 MPa and 283 K (E) and for TIP4P/Ice at 50 MPa and 293 K (F).

* Corresponding author:letizia.tavagnacco@cnr.it

† Corresponding author:emanuela.zaccarelli@cnr.it

‡ Corresponding author:ester.chiessi@uniroma2.it

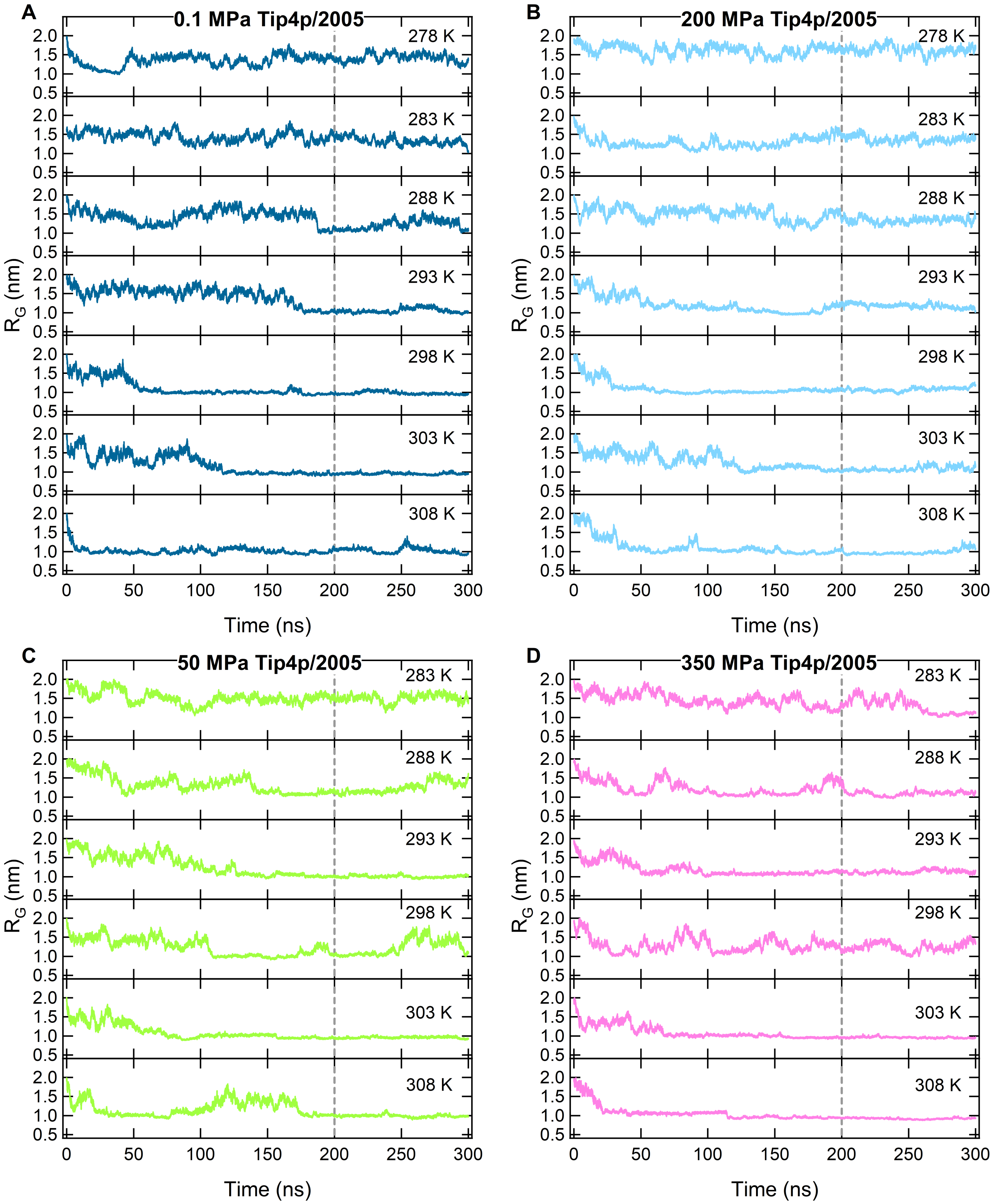

FIG. S2. Time evolution of PNIPAM radius of gyration in TIP4P/2005 as a function of temperature at pressure values of (A) 0.1 MPa, (B) 200 MPa, (C) 50 MPa, and (D) 350 MPa.

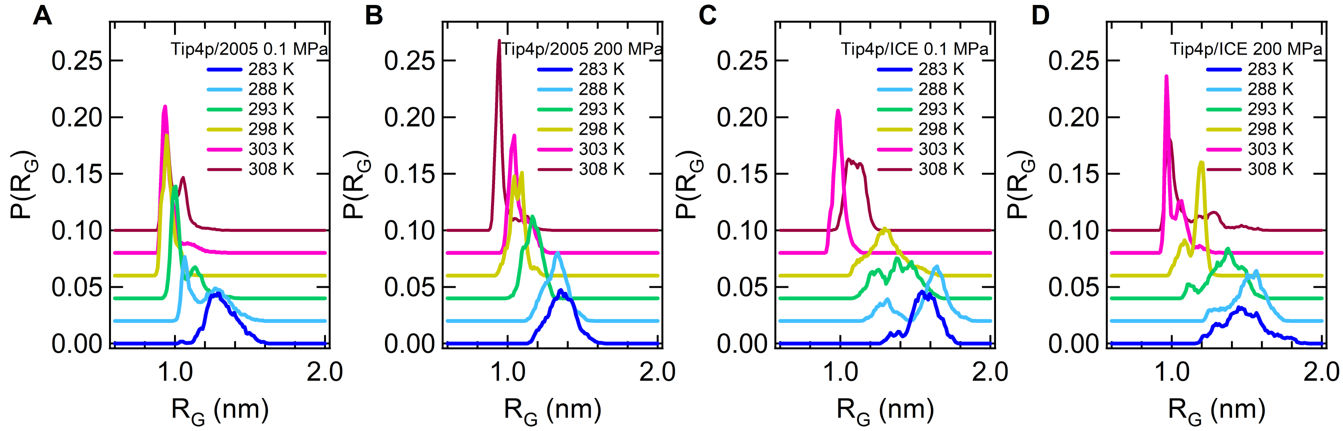

FIG. S3. Distribution of the radius of gyration P(R_G) of the polymer chain as a function of temperature at pressure values of 0.1 MPa and 200 MPa. Data refer to Tip4p/2005 simulations (A and B) and Tip4p/ICE simulations (C and D).

II. HYDRATION OF PNIPAM IN TIP4P/2005 AND TIP4P/ICE

Figure S4 displays the correlation between R_G and the total number of hydration water molecules at 283 K. The results reveal that the smaller R_G values sampled in TIP4P/2005 water are related to a lower hydration degree of the polymer chain. The effect of pressure on the hydration properties of PNIPAM in TIP4P/2005 and TIP4P/Ice was characterized by calculating the temperature dependence of the number of hydration molecules at constant pressure values of 0.1, 50, 200, and 350 MPa, as reported in Figure S5. The degree of hydration of PNIPAM increases in the high pressure regime, as compared to the atmospheric pressure, in both water solutions.

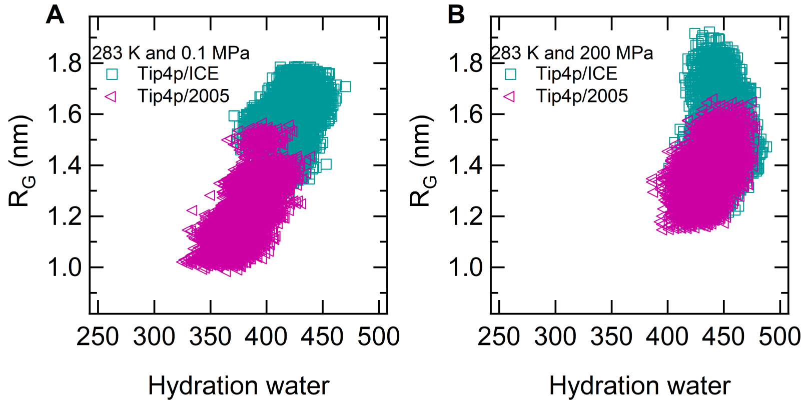

FIG. S4. Correlation between PNIPAM radius of gyration and number of hydration water molecules at pressure values of (A) 0.1 MPa and (B) 200 MPa for TIP4P/Ice (green squares) and TIP4P/2005 (violet triangles).

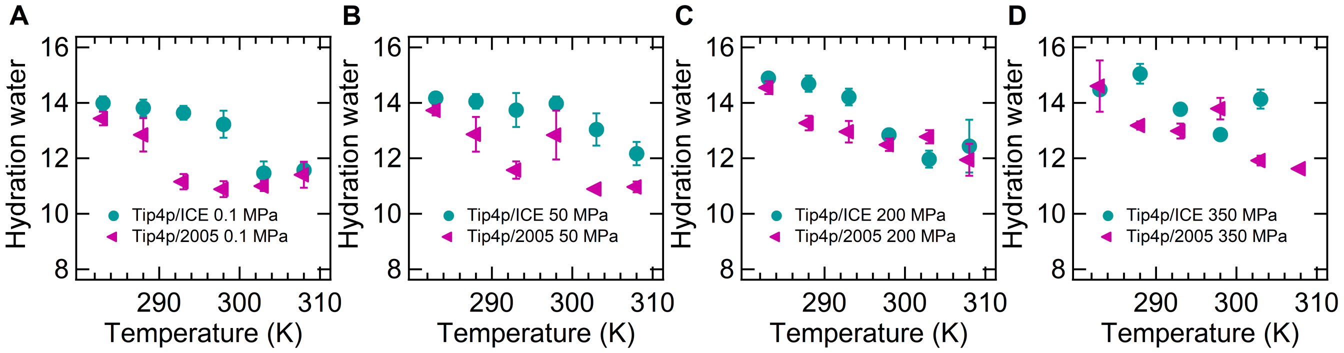

FIG. S5. Temperature dependence of the total number of hydration water molecules normalized to the number of repeating units and averaged over the last 100 ns of simulation. Data are calculated at (A) 0.1 MPa; (B) 50 MPa; (C) 200 MPa; and (D) 350 MPa for TIP4P/Ice (green circles) and TIP4P/2005 (violet triangles).

III. PNIPAM-TIP4P/2005 PHASE DIAGRAM DATA

To build PNIPAM pressure-temperature phase diagram, the average radius of gyration of the polymer chain was used as parameter to define state points where globule or coil state are more stable. The results are summarized in Table S1. Globular states were assigned to conformations with an average radius of gyration smaller than 1.2 nm.

TABLE S1. Average chain radius of gyration in TIP4P/2005.

T (K)	0.1 MPa	30 MPa	50 MPa	100 MPa	200 MPa	350 MPa
283	(1.33±0.10)	(1.42±0.11)	(1.51±0.11)	(1.55±0.13)	(1.32±0.13)	(1.30±0.19)
288	(1.22±0.13)		(1.25±0.15)	(0.99±0.03)	(1.44±0.16)	(1.10±0.06)
293	(1.05±0.06)		(1.00±0.03)	(0.97±0.01)	(1.20±0.18)	(1.13±0.05)
298	(0.98±0.04)		(1.24±0.21)		(1.09±0.16)	(1.24±0.11)
303	(0.95±0.02)	(0.98±0.09)	(0.96±0.02)	(1.17±0.07)	(1.22±0.20)	(0.96±0.02)
308	(1.03±0.07)	(1.13±0.07)	(0.99±0.03)	(1.00±0.05)	(1.08±0.20)	(0.92±0.02)

Radius of gyration averaged over 100 ns with standard deviation.
